# Visualizing simultaneous eye-hand movements in a MATLAB dashboard

**DOI:** 10.1016/j.mex.2024.102722

**Published:** 2024-04-30

**Authors:** Hudson TE, Seiple A, Shafiee Sabet A, Beheshti M, Rizzo JR

**Affiliations:** a Department of Rehabilitation Medicine, NYU Langone Health, 244 E38th St., New York, NY, 10016, USA; b Department of Neurology, NYU Langone Health, 222 E40th St., New York, NY, 10016, USA; c Department of Biomedical Engineering, NYU Tandon School of Engineering, 433 First Avenue, New York, NY, 10010, USA; d Department of Mechanical & Aerospace Engineering, NYU Tandon School of Engineering, 433 First Avenue, New York, NY, 10010, USA

**Keywords:** *Eye-Hand Dashboard*, Sensory-motor coordination, Visualization techniques, Eye tracking, Limb tracking

## Abstract

Eye-hand coordination (EHC) is crucial to our daily activities, and its underlying mechanisms are being intensely studied. The analysis of simultaneous eye and hand movements can provide valuable insights into EHC, particularly for individuals struggling with dexterous control, such as might be caused by stroke or traumatic brain injuries. Despite advancements in motion-capture and eye tracking technologies, there is currently no automated method for visualizing concurrent eye- and hand-movement data. To address this need, we have developed a MATLAB-based dashboard designed for near instantaneous analysis and visualization of eye and hand-tracking data. This paper introduces the design of the dashboard and presents experimental results obtained from its application, leveraging simulated data inspired by our recent work in stroke. This testing suggests that our solution has the potential to significantly aid in understanding and investigating EHC by providing side-by-side and time-locked comparison of eye/hand movements along with their timing and spatio-temporal errors, offering novel opportunities for research and clinical applications.

• Continuous eye movement data is collected throughout the experiment
• Continuous hand movement data is collected throughout the experiment
• Combine datasets and display time-locked eye-hand data in a single dashboard

## Graphical abstract

**Figure:**
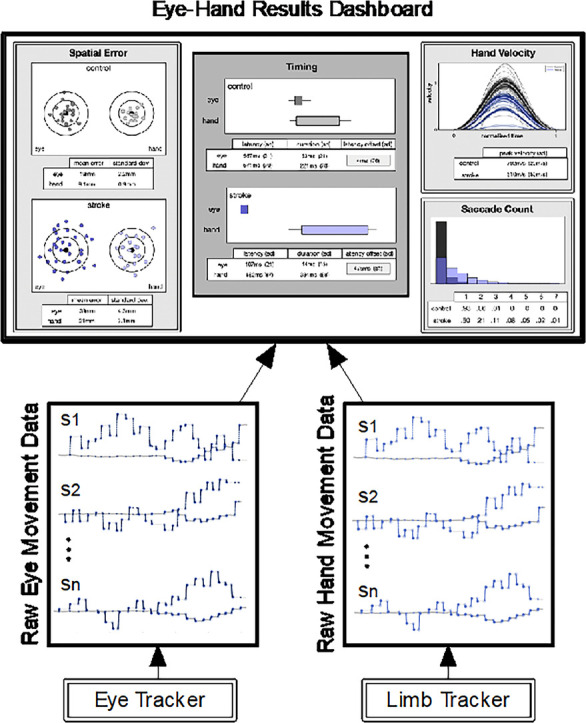

Specifications table

**Table:** 

Subject area:	Neuroscience
More specific subject area:	*Eye-Hand Coordination*
Name of your method:	*Eye-Hand Dashboard*
Name and reference of original method:	*Rizzo JR, Fung JK, Hosseini M, Shafieesabet A, Ahdoot E, Pasculli RM, Rucker JC, Raghavan P, Landy MS, Hudson TE. Eye Control Deficits Coupled to Hand Control Deficits: Eye-Hand Incoordination in Chronic Cerebral Injury. Front Neurol. 2017;8:330.*https://doi.org/10.3389/fneur.2017.00330
Resource availability:	*NA.*

## Method details

Eye-hand coordination (EHC) is crucial for daily activities like food preparation and is a key focus in rehabilitation and movement disorders research. Coordinated eye and hand movements involve intricate interactions between neurological subsystems, and disruptions caused by events like strokes or neurodegenerative diseases significantly impact EHC [[1], [2], [3], [4], [5]]. With the advent of video-based eye-tracking technologies, monitoring these movements has become feasible. However, existing software lacks the capability to concurrently analyze eye and hand movement data. There is a clear need for automated analysis and visualization tools that integrate data from both systems, emphasizing spatiotemporal dependencies and relationships.

We designed a custom, integrated eye-hand analysis pipeline that displays/visualizes relevant metrics in a ‘dashboard’-style interface, easing barriers to use and comprehension. The primary dashboard will present a report-card-like evaluation portal with four main metrics: spatial error (of eye and hand), timing and latency (of eye and hand), number of saccades (eye), and a velocity profile (hand). Within this visual representation, we provide direct measures of integrated eye-hand performance or spatiotemporal registration for relevant effectors to the patient and clinician.

Equipment

1) Eye Tracking equipment would typically include a head-mounted (e.g., Pupil Core, made by Pupil Labs [6]), or tabletop-mounted (e.g.,. EyeLink 1000+, made by SR Research [REF]) camera for recording eye movements. Such systems allow for accurate monitoring of pupil size and location even when faced with potential obstructions, such as eyelashes [[6], [7], [8]].
2) Hand Tracking can be accomplished via several methods, including optical (e.g., Optotrak) and electromagnetic (e.g., Polhemus LIBERTY) tracking systems. These limb tracking systems typically include multiple sensors, and acquire data at >200 Hz. These systems deliver an uninterrupted data stream for high-fidelity tracking that can be implemented independently, in a way that will not interfere with the operation of the eye tracking equipment.

Notably, the dashboard takes the time series of eye and hand positions (along with target position) as input, it is hardware-agnostic, making it universally applicable across data acquisition platforms.

## Data acquisition and processing

Data can generally be collected, saved, and viewed using software native to each tracking system. For example, Pupil Labs provides plugins written in Python and C, and can record video from the two eye cameras and also the world-view camera, allowing for an interactive view of gaze information overlaid on environmental video. The system also allows for playback and export of these videos. Their pupil detection algorithm features Canny filtering, and a combinatorial search through edges that meet curvature and continuity criteria [9]. The detection begins upon eye recognition and is streamed to the interface, which is supported on Linux, MacOS, and Windows [6].

## The EHC Dashboard

**Fig. 1:**
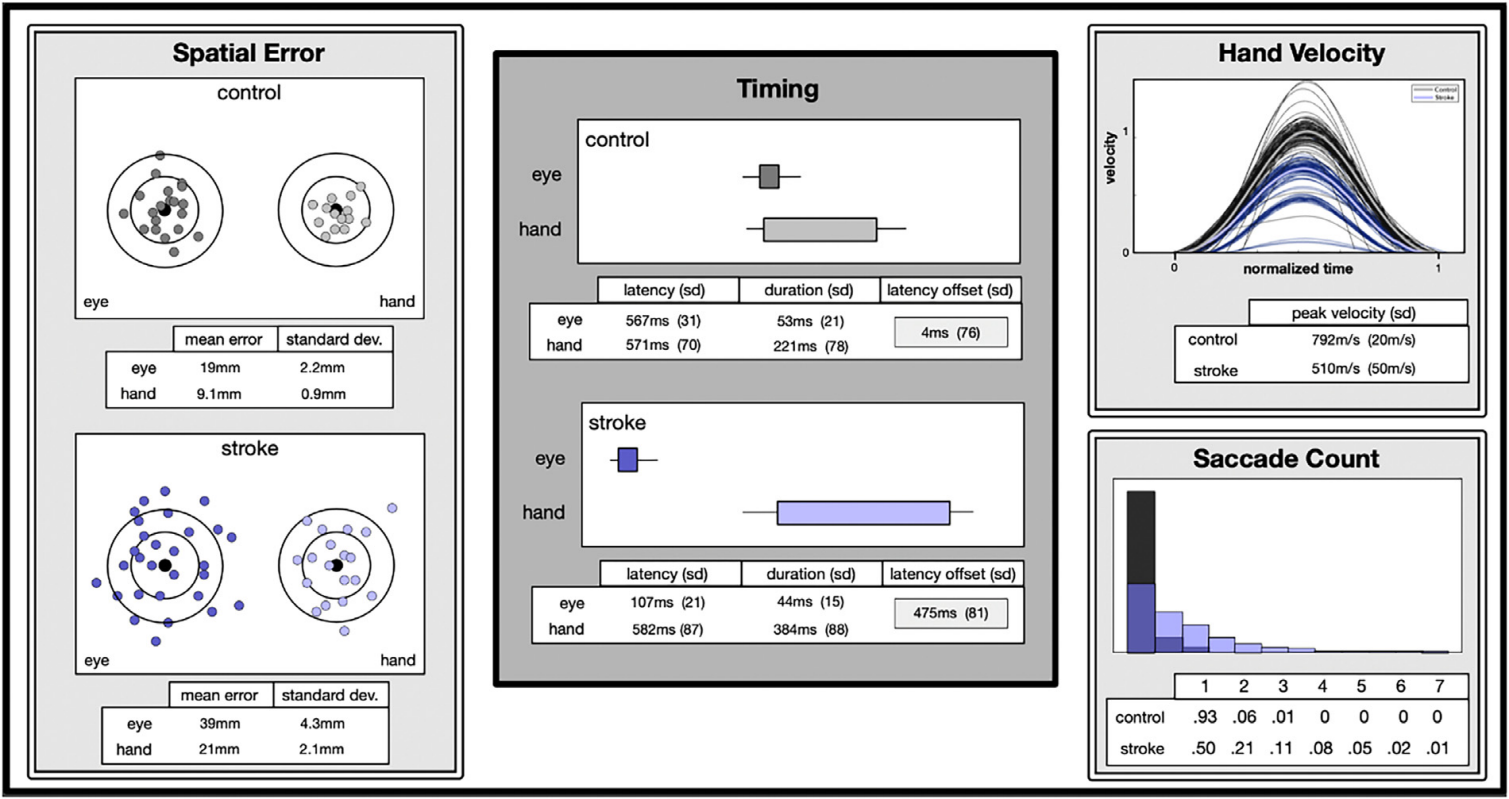
Here is an example of our methodology using eye and hand movement data collected using Pupil Core and Polhemus limb tracker.

The report card visualization provides a novel way to view and analyze these complex behaviors in a user-friendly, efficient, and thorough manner. To optimally extract the information present in this type of experimental data, the dashboard provides a variety of metrics for both eye and hand movements. It contains four main components: spatial error, timing, hand velocity, and saccade count [Fig. 1]

• The spatial error is shown as four scatterplots of saccadic/reach endpoints relative to the target position.
• Timing information, including movement onset, offset times, and duration, is presented as boxplots in the middle column. The start and end of each box indicate average movement onset and offset times, with the average duration given by box width. Latency offset (defined as the difference between hand and eye movement onset), which provides a measure of eye-hand temporal coordination, is given in each group's timing table (bottom).
• The hand velocity plot displays time-normalized velocity profiles scaled to the average peak velocity of the control group. Average velocity traces are plotted as thick lines and individual movements as thin lines in the same color.
• The saccade count section provides histograms of the number of saccades made, per trial, by each group. A typical control subject will generally either make just one saccade to the target or one main and one corrective saccade on each trial; participants with neurological impairment tend to make many more [10].

The ease of this visualization may allow healthcare professionals to interpret research results faster, recognize trends, easily compare patient data against controls, and make more informed decisions [[11], [12]]. Additionally, clinicians can use report cards to estimate recovery timelines based on patients with comparable results [[11], [13]].

From a rehabilitation perspective, access to visualizations that clearly display a patient's progress can be used as positive reinforcement and significantly aid the recovery process. Quantifying progress under various circumstances, such as before or after physical therapy, could motivate better outcomes while improving access to medical information.

## Method validation

We simulated data based on a study of 32 subjects, 17 control (age 26.2 ± 4.6y, male 29 %) and 15 stroke participants (age 55.8 ± 14y, male 53 %) [1]. All stroke participants had a history e of middle cerebral artery (MCA) cerebrovascular accident (demographics summarized in Table 1). The subjects participated in an experiment involving look-and-reach tasks. This study was approved by the Institutional Review Board of New York University's School of Medicine. Written informed consent was obtained from all participants.

**Table 1:** Demographics.

Subject ID	Age (Years)	Gender
1	78	Male
2	61	Female
3	34	Male
4	39	Female
5	70	Male
6	60	Female
7	73	Male
8	51	Female
9	60	Male
10	39	Male
11	70	Male
12	47	Female
13	65	Female
14	50	Male
15	40	Female

## Results

The report card generated from the dashboard (refer to Fig. 1) provides a comprehensive overview of the study results, as summarized in Table 2. Spatial error emerged as a notable distinction between stroke participants and controls, with stroke individuals exhibiting significantly larger reach endpoint errors and increased saccade errors (mean and standard deviation of endpoint-target deviation). The average primary saccade initiation time (latency) was considerably shorter in stroke patients than control subjects. Stroke patients also began reaches slightly later (mean latency). Control movements were characterized by coupled saccade and reach onsets. Temporal decoupling occurs when the time between the primary saccade and the beginning of a reach is prolonged, breaking the stereotyped timing of paired target-image acquisition and arm movement. The control group had, on average, a single primary saccade, while stroke subjects were much more likely to have made multiple saccades during a trial. Temporally coordinated eye-hand movements are characterized by saccades that are ‘held’ until reach completion, as demonstrated by the control group. The temporal dyscoordination exhibited by stroke participants occurred in part because their average initial saccade was made very early, and in part by retarded limb movements, increasing by two orders of magnitude the latency offset between saccade and reach.

**Table 2:** Collected results.

	Stroke [SD]	Control [SD]
Saccade delay	107 ms [21 ms]	567 ms [31 ms]
Reach delay	582 ms [87 ms]	571 ms [70 ms]
Latency Offset	475 ms [81 ms]	4 ms [76 ms]
Saccade endpoint error	39 mm [4.3 mm]	19 mm [2.2 mm]
Reach endpoint error	21 mm [2.1 mm]	9.1 mm [0.9 m]

## Future work

Future work for the dashboard will focus on adding enhanced/secondary configuration options and clickable expansions of the plot sections. For example, we plan to expand the Hand Velocity section with a main sequence plot for clinicians concerned about the possibility of slow saccades. In addition, we plan to make each section clickable, for expanded analysis. For example, one might click on the timing tab to move to a screen that includes only timing data displayed in different ways (such as eye movement timing relative to the hand and vise veras, and raster plots of saccadic occurrence [10]), or one might click on the error section to move to a screen entirely focused on error plots given in different ways (eye relative to hand error, distance error, anglular error, etc.). As the dashboard evolves, figuring out the best way to help clinicians understand how to use its features and navigate its interface will becomes a crucial aspect. Improving the visualizations and adding more cues to visualize patient performance could increase use and aid in training/recovery. For example, the ability to pull up previous report cards and compare these to the current performance, allowing clinicians to quantify progress under various circumstances, such as before or after physical therapy, will be an important use of this dashboard.

## CRediT authorship contribution statement

**Hudson TE:** Conceptualization, Methodology, Formal analysis, Writing – review & editing, Visualization, Supervision. **Seiple A:** Data curation, Formal analysis, Software. **Shafiee Sabet A:** Writing – original draft. **Beheshti M:** Writing – review & editing, Supervision, Project administration. **Rizzo JR:** Conceptualization, Writing – review & editing, Visualization, Supervision.

## Declaration of competing interest

The authors declare that they have no known competing financial interests or personal relationships that could have appeared to influence the work reported in this paper.

## Data Availability

No data was used for the research described in the article.
